# A Systematic Review: State of the Science on Diagnostics of Hidden Hearing Loss

**DOI:** 10.3390/diagnostics15060742

**Published:** 2025-03-16

**Authors:** Sunil Shenoy, Khushi Bhatt, Yalda Yazdani, Helia Rahimian, Hamid R. Djalilian, Mehdi Abouzari

**Affiliations:** Division of Neurotology and Skull Base Surgery, Department of Otolaryngology-Head and Neck Surgery, University of California, Irvine, CA 92697, USA

**Keywords:** hidden hearing loss, synaptopathy, auditory brainstem response, middle ear muscle reflex, electrocochleography, frequency-following response

## Abstract

**Background/Objectives**: A sizeable population of patients with normal pure-tone audiograms endorse a consistent difficulty of following conversations in noisy environments. Termed hidden hearing loss (HHL), this condition evades traditional diagnostic methods for hearing loss and thus is significantly under-diagnosed and untreated. This review sought to identify emerging methods of diagnosing HHL via measurement of its histopathologic correlate: cochlear synaptopathy, the loss of synapses in the auditory nerve pathway. **Methods**: A thorough literature search of multiple databases was conducted to identify studies with objective, electrophysiological measures of synaptopathy. The PRISMA protocol was employed to establish criteria for the selection of relevant literature. **Results**: A total of 21 studies were selected with diagnostic methods, including the auditory brainstem response (ABR), electrocochleography (EcochG), middle ear muscle reflex (MEMR), and frequency-following response (FFR). Measures that may indicate the presence of synaptopathy include a reduced wave I amplitude of ABR, reduced SP amplitude of EcochG, and abnormal MEMR, among other measurements. Behavioral measures were often performed alongside electrophysiological measures, the most common of which was the speech-in-noise assessment. **Conclusions**: ABR was the most common diagnostic method for assessing HHL. Though ABR, EcochG, and MEMR may be sensitive to measuring synaptopathy, more literature comparing these methods is necessary. A two-pronged approach combining behavioral and electrophysiological measures may prove useful as a criterion for diagnosing and estimating the extent of pathology in affected patients.

## 1. Introduction

Hearing loss (HL) is the most common sensory impairment worldwide, with an estimated 1 in 5 Americans suffering from some degree of HL [1,2]. The presence of HL increases the risk of developing several comorbidities, including cognitive decline, mobility impairment, social isolation, and poor wellbeing, all of which significantly reduce quality of life [3,4]. With an estimated 72 million people affected in the U.S. and hundreds of millions of people affected abroad, HL is a critical public health issue, and it is important to be able to identify accurate methods of its diagnosis [5]. Given HL’s diverse etiologies and varied clinical presentation, knowledge of current diagnostic methods will allow healthcare providers to better understand the origin of patients’ symptoms and determine corresponding options for treatment.

Audiometry is considered the cornerstone for HL diagnosis, as it is relatively simple, efficient, and aids in determining the presence and extent of hearing impairment for the patient [6]. Pure-tone audiometry (PTA) involves the delivery of varying intensities (10–120 dB) of sound over a range of different frequencies (250–8000 Hz) and assessing patient response to each stimulus [7]. Results are graphed to produce an audiogram, which establishes the severity of the patient’s hearing deficit and minimum threshold of hearing. Permanently elevated auditory thresholds are indicative of sensorineural HL (SNHL). A PTA can further be leveraged to assess bone conduction with an oscillator placed over the mastoid process, measuring threshold frequencies via direct stimulation of the cochlea, bypassing the outer and middle ear structures [8]. This technique allows for a direct comparison of air and bone conduction to test for a potential “air-bone gap”, which indicates a conductive or mixed HL rather than the more common SNHL [9].

However, there is a sizeable population of patients with normal pure-tone audiograms who describe their hearing loss as a consistent difficulty following conversations in noisy environments [10,11]. Because these patients endorse significant perceptual abnormalities with seemingly normal auditory sensitivity, this condition has been termed “hidden hearing loss” (HHL) [12]. Additional symptoms expressed by HHL patients include struggling to follow conversations and a subjective lack of sound clarity, with consequential mental fatigue [13]. Identified risk factors in the development of HHL are noise exposure, ototoxic drugs, peripheral neuropathies, and aging—all of which commonly impact SNHL as well [14,15]. Prominent comorbidities of HHL include tinnitus and hyperacusis, with HHL also serving as a risk factor for more severe HL pathology, such as SNHL with permanently elevated auditory thresholds [13,16]. Due to obvious barriers in the diagnosis of HHL, its prevalence is not entirely parsed out, though preliminary studies estimate between 10 and 12% of patients with HL specifically suffer from HHL [17,18]. With a significant amount of likely undiagnosed and untreated patients, proper diagnostic tools are necessary to identify symptomatic patients and reduce the risk of disease progression.

The pathophysiology of HHL relies on its histopathologic correlate, cochlear synaptopathy, defined as the loss of synapses in the auditory nerve pathway [19,20]. Until recently, it was long considered that peripheral cochlear hair cells are much more vulnerable to damage than cochlear nerve fibers [21]. This is likely due to several animal model studies that found evidence of hair cell damage within hours of insult with noise or ototoxic drugs, whereas damage to the spiral ganglion cells’ (SGCs’) structures occurred in the following months [22,23,24]. Hair cell damage and apoptosis translate to elevated threshold values on audiometry assessment and suggest evidence of SNHL. Recent studies, however, have found that hair cell damage does not necessarily precede damage to nerve fibers [21]. In fact, in noise-induced and age-related HL, afferent fibers between hair cells and spiral ganglion neurons (SGNs) are most vulnerable to damage, with these “cochlear ribbon synapses” degenerating first [20,25,26]. Ribbon synapses, located in the basilar aspect of inner hair cells, are a pivotal part of the auditory pathway as they prime vesicles for glutamate release into the synaptic cleft, eventually activating SGCs and translating auditory signals to the brainstem [27]. Older studies were likely unable to identify synaptopathy as a major driver of HHL because hair cells and SGNs retain their normal morphology for months to years despite the damage to the synapse [28]. More recent papers have also identified that excessive noise and demyelinating diseases can damage the myelin sheath of the cochlear nerve fibers, inducing HHL with normal audiograms [29]. Regardless of etiology, this intermediate state of afferent peripheral nerve damage and temporary hair cell structure preservation potentially explains the speech discernment symptoms of HHL amidst its normal audiometry findings. Furthermore, these findings suggest that HHL likely increases risk of progression to SNHL following continued aging, disease processes, or ototrauma.

There have been a handful of proposed methods for measuring synaptopathy, particularly involving either electrophysiological activity or behavioral measures. One of the most predominant electrophysiological measures includes the auditory brainstem response (ABR), which involves quantifying neural activity along the auditory pathway following an acoustic stimulus [30]. The wave I amplitude of the ABR is of particular interest in measuring synaptopathy as it reflects the initial activity of the auditory nerve following noise exposure, which might demonstrate an impaired response due to loss of hair cell nerve fibers [31]. Another emerging method of measurement is the middle ear muscle reflex (MEMR). Given that the MEMR relies on the auditory nerve to transmit high-threshold potentials to the brainstem, a dampened reflex could indicate synaptopathy [32]. In contrast to these electrophysiological measures, subjective behavioral responses have also been utilized to assess HHL. A common example is the speech-in-noise (SiN) test, which evaluates the patient’s ability to understand and identify spoken words in background noise [33]. While not objective, SiN and other behavioral measures can provide insight into the extent of symptom severity for HHL patients.

As HHL affects a significant portion of those with HL and evades traditional methods of screening, identification of diagnostic tools capable of HHL detection is imperative for early intervention and treatment. With no universally agreed upon criteria for HHL diagnoses, comparisons between potential methods are necessary to develop a reliable diagnostic assessment. In this review, we further discuss the current diagnostic measures utilized for detecting HHL and how they correspond with disease pathophysiology. We also identify future directions for research and discuss potential standardization strategies for diagnoses.

## 2. Materials and Methods

This study was conducted based on the Preferred Reporting Items for Systematic Reviews and Meta-Analyses (PRISMA) protocol (Supplementary Materials). The steps followed included initial study design, literature search of databases, collection of articles, screening based on article content, and evaluation of relevant articles. Risk of bias within the selected studies was evaluated via the Joanna Briggs Institute (JBI) critical appraisal checklist for systematic reviews investigating diagnostic test accuracy.

### 2.1. Search Strategy

Several online library databases were electronically queried, including PubMed, Cochrane Library, Google Scholar, and SCOPUS. The search sought to gather literature detailing diagnostic methods for assessing HHL and/or cochlear synaptopathy, with the purpose of comparing the various tools in terms of reliability, methodology, and translation to clinical use. The relevant Medical Subject Headings (MeSH) terms used for the search are as follows: “(hidden hearing loss OR cochlear synaptopathy OR cochlear neuropathy) AND (diagnosis OR measure OR detection)”; and “(hidden hearing loss) AND (synapse OR synaptopathy) AND (diagnosis OR measure OR detection)”. The keywords searched included: hidden hearing loss, cochlear synaptopathy, subclinical hearing loss, auditory neuropathy, and cochlear neuropathy. Articles were selected and screened on the basis of title, abstract, and content, with removal of duplicates and automated filtering of ineligible papers. All articles were published in peer-reviewed journals.

### 2.2. Inclusion Criteria

Original studies published in or after 2019, which included analyses investigating the performance of diagnostic methods determining the presence and possible extent of HHL/cochlear synaptopathy, were included as interventions for this review. This restriction to papers published in or after 2019 was implemented, because HHL was newly introduced as a concept in 2009, and as such, diagnostic modalities have only recently been introduced. Older studies have the potential to lack the sensitivity and specificity of newer diagnostic methods, which is important for conditions such as HHL and cochlear synaptopathy that are more novel and in which our understanding of them is evolving. Such diagnostic techniques must have measured at least one physiological outcome (as opposed to a purely behavioral or perceptual measurement) with objective, quantifiable data. This study’s outcomes sought to assess the performance and reliability of these diagnostic methods for detecting HHL/cochlear synaptopathy. Physiological outcomes are defined as those that measure a biological response, such as neural activity or reflex presence, with minimal or no active participation from the subject. Only human studies with adult subjects were considered. Our study’s population included all participants from studies who demonstrated normal auditory sensitivity (≤25 dB) up to 8 kHz with consequent normal audiograms. Studies must also have a clearly defined at-risk/symptomatic group and healthy/low-risk control group to allow for direct comparison of diagnostic methods between groups. Those considered in the at-risk group must have demonstrated either (a) symptomatic endorsement of HHL, (b) increased risk of HHL due to long-term noise exposure, or (c) age-related risk of HHL to merit inclusion. The healthy/low-risk control group was defined as having normal audiograms and a comparatively reduced risk of HHL due to factors such as age or lower noise exposure.

### 2.3. Exclusion Criteria

Studies that did not include a diagnostic framework and attempted the assessment of HHL and/or cochlear synaptopathy were excluded. Studies using animals or utilizing animal models were not considered. Experiments assessing populations with clinically significant audiometric thresholds and/or the diagnosed presence of SNHL, presbycusis, or other otological conditions were excluded. Experiments that utilized purely subjective measures of synaptopathy, such as speech-in-noise tests or other behavioral tests, with a lack of physiological measures, were excluded. Correlational studies that lacked separate, clearly defined at-risk and control groups were excluded. Case reports, commentaries, reviews, and letters to the editor were excluded.

### 2.4. Summary of Selection

**Figure 1:**
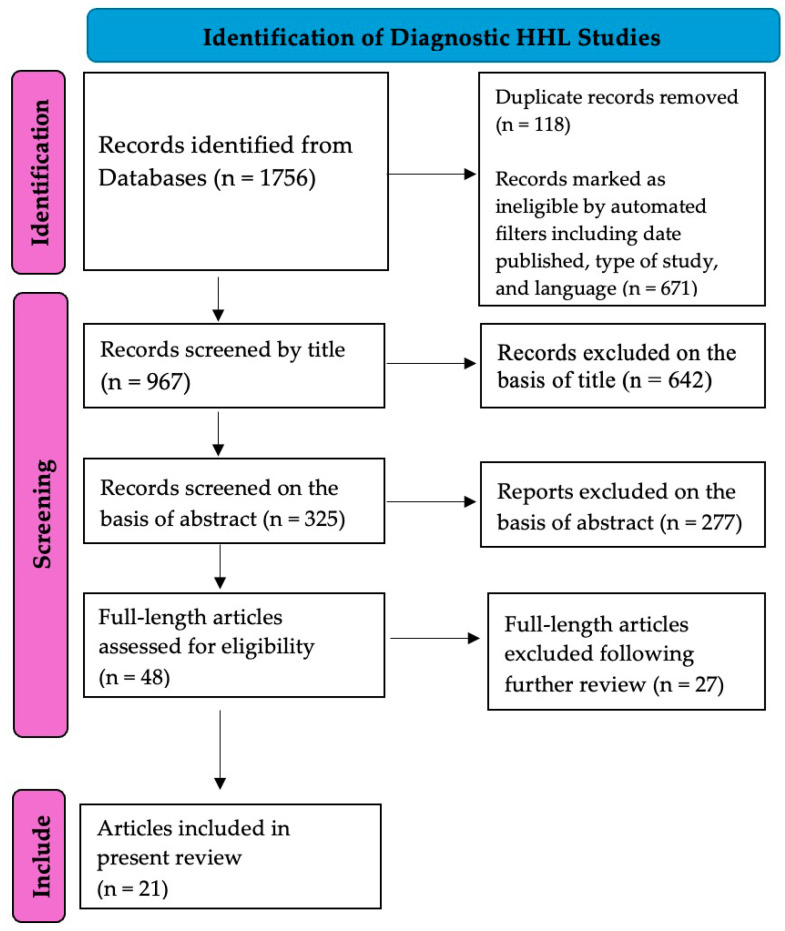
PRISMA flow diagram illustrating the retrieval and screening process of relevant articles (PRISMA citation) [61].

Figure 1 below displays the PRISMA flowchart with respect to the selection process of articles for this review. The selection process began on 3 January 2025 and was completed by 25 January 2025. A secondary verification of the selected papers and manuscript drafting also occurred during this period. In summary, a total of 1756 articles were identified throughout the available databases on preliminary search. A total of 118 articles were removed due to duplicate articles. The remaining articles were screened by title and abstract by two authors independently, with a total of 21 studies found to be relevant to this review. Table 1 below includes the names of the studies excluded during the final step of the PRISMA selection process and their reasons for exclusion. For each study, its diagnostic method, subject population, behavioral correlate (if applicable), and any key findings were sought, which is summarized in Table 2. Inter-judge selection was utilized to validate chosen articles, reduce bias when selecting studies, and evaluate potential bias within eligible studies, with disputes being discussed among authors.

**Table 1:** Excluded articles and corresponding reason for exclusion.

Author (Year)	Reason for Exclusion from Review
Valderrama et al. (2018) [34]	Evaluation of a correlational relationship between lifetime noise exposure and ABR, rather than two distinct at-risk and control groups. The study was also released prior to 2019.
Guest et al. (2019) [35]	Assessed MEMR thresholds exclusively in subjects with tinnitus, rather than noise exposure or age. The presence of previously identified otologic conditions such as tinnitus merit exclusion.
Kramerer et al. (2019) [36]	The study sought to identify the effects of cognitive capacity in influencing physiologic and behavioral measures of CS. It focused on the assessment of variance in measuring synaptopathy and identifying confounding variables rather than quantifying synaptopathy between groups.
Prendergast (2019) [37]	This study included the use of a regression model to determine relative contributions of age and noise exposure on synaptopathy. There was a lack of clearly defined at-risk and control groups.
Marmel et al. (2020) [38]	Assessed CS in tinnitus sufferers only. Not all participants demonstrated normal audiograms.
Megarbane and Fuente (2020) [39]	The sample included participants who did not have occupational noise exposure; no stratification of groups by relative risk of CS.
Mepani et al. (2020) [40]	Assessment of purely normal-hearing subjects with no stratification of group by risk, with correlational analyses.
Nam et al. (2020) [41]	The use of acute noise exposure to identify at-risk patients, rather than long-term or lifetime noise exposure.
Bal and Derinsu (2021) [42]	Several participants in this study did not display normal pure-tone audiograms.
Chen et al. (2021) [43]	The study assessed the presence of CS in patients already diagnosed with presbycusis, rather than participants with no previously identified ear pathology.
Wang et al. (2021) [44]	Measurements of CS occurred following acute noise exposure (attendance of a music festival) rather than long-term noise exposure.
Carcagno and Plack (2022) [45]	The study assessed whether variance in masked-speech reception tasks can be explained by the variance in ABR and FFR. No assessment of diagnostic value for CS.
Kaf et al. (2022) [46]	Use of acute noise exposure (6 months) to stratify high- and low-risk groups, rather than long-term exposure.
Lobdell et al. (2022) [47]	Correlational study design assessing the reliability of MEMR between laboratory and clinical measures.
Goodman et al. (2023) [48]	The study provides a recommended study design and protocol for using ECochG to measure CS to reduce variability in measurements.
Haggerty et al. (2023) [49]	Use of animal models (gerbils) to assess CS rather than human subjects.
Shehabi et al. (2023) [50]	Association study design determining the relationship between MEMR and binaural temporal coding performance. Lack of defined at-risk and control groups.
Couth et al. (2024) [51]	Cumulative levels of noise exposure between the control and at-risk group were similar, indicating no difference in susceptibility in measuring CS.
De Poortere et al. (2024) [52]	The study measured intra-subject variability in measures of CS to assess the reliability of these measures and possible learning effects. Lack of diagnostic measurements for CS.
Ding et al. (2024) [53]	Assessed correlation between pure-tone audiometry and DPOAEs. Participants with abnormal audiograms were also included.
Fujihira et al. (2024) [54]	Correlational study design with lack of control and at-risk groups.
Kamerer et al. (2024) [55]	The study assessed the percentage of variance in hearing thresholds explained by physiological and behavioral measures of HHL.
Liu et al. (2024) [56]	The study involved the development of a novel model quantifying perceptual hearing consequences due to cochlear deafferentation. Lack of assessment of CS in human subjects.
McFarlane and Sanchez (2024) [57]	Correlational study design with no clearly defined at-risk and control groups.
Saade et al. (2024) [58]	Correlational study design assessing extended high-frequency thresholds with subjective audiologic symptoms.
Schirmer et al. (2024) [59]	The study did not exclude participants with abnormal audiograms.
Temboury-Gutierrez (2024) [60]	Participants included in this study showed normal audiograms between 125 Hz and 4 kHz, rather than up to 8 kHz.

**Table 2:** Selected articles arranged chronologically with relevant information pertaining to diagnostic methods, subjects, behavioral measures (if performed), and key findings.

Author (Year)	Diagnostic Method	Subject Population	Behavioral Correlate (If Applicable)	Key Findings
Bhatt and Wang (2019) [62]	Click-evoked auditory brainstem response (ABR)	32 females; 18 with high noise exposure background (NEB) and 14 with low NEB.	Dichotic listening performance, speech-in-noise (SiN) performance	No significant difference in ABR wave I, III, and V amplitude between high and low NEB groups. No significant difference in speech-in-noise performance. Higher NEB participants demonstrated worse dichotic listening performance.
Carcagno and Plack (2020) [63]	Ratio of ABR wave I amplitude at high and low click levels (WI_H_/WI_L_), and difference in frequency-following response (FFR) at shallow and deep-modulated tones (FFR_S_-FFR_D_). Utilized high-pass masking to exclude response from high- frequency cochlear regions.	102 individuals, separated into three age groups: young (*n* = 34), middle-aged (*n* = 34), and older (*n* = 34).	None	WI_H_/WI_L_ ratio declined with age without high-pass masking, though it did not decrease in the masking condition. FFR components including temporal fine structure (TFS) and envelope (ENV) decreased with age. However, no age-related decreases of FFR_S_-FFR_D_ were found with and without masking. Electrophysiological measures assessing purely low-frequency regions may not be a valuable measure of cochlear synaptopathy.
Dewey et al. (2020) [64]	Click-evoked auditory brainstem response (ABR), and fMRI of ascending auditory pathway.	Individuals aged 25–40, separated by lifetime noise exposure: high (*n* = 32) and low (*n* = 30).	None	Participants in the high noise exposure group had significantly enhanced fMRI responses to broadband noise compared with the low exposure group, suggesting central hyperactivity. Sustained responses did not differ among groups. No significant group differences were found in the ABR response between groups, including amplitudes of wave I and V, as well as the ratio of wave I/V.
Kikidis et al. (2020) [65]	ABR response amplitudes and latencies for waves I, II, and V, measured at 3 click rates (11/s, 33/s, and 44/s)	Two groups, aged 18–41: 24 musicians with occupational noise exposure and 24 healthy controls.	Speech in bubble audiometry	Wave I amplitude was significantly lower among the musician experimental group at all 3 click rates. Also, the experimental group demonstrated a significant reduction in the wave I/V ratio for the 33/s click rate.
Bal and Derinsu (2021) [42]	Tympanometry, electrocochleography (EcochG), ABR	50 healthy young adults stratified by total noise level from personal devices. High-risk (*n* = 25) and low-risk (*n* = 25) groups.	Matrix Test	The action potential (AP) amplitude was significantly decreased in EcochG in high-risk compared with low-risk subjects. Wave V of ABR was also significantly decreased between groups. High-risk participants performed worse on the matrix test.
Bramhall et al. (2021) [66]	ABR wave I amplitude and envelope following response (EFR)	Young individuals (aged 19–35). There were 2 groups, consisting of veterans with a history of noise exposure and healthy non- veterans.	None	There was a reduction in the EFR magnitude and ABR Wave I amplitude among the Veteran population compared to the non-Veterans.
Megha et al. (2021) [67]	ABR, distortion product otoacoustic emissions (DPOAEs), contralateral suppression of OAE (CSOAE)	60 adult males. Group 1: 20 subjects with no noise exposure. Group 2: 20 subjects aged 45–65 with no occupational noise exposure. Group 3: 20 subjects < 35 years old exposed to occupational noise.	None	Group 1 demonstrated greater CSOAEs compared with Groups 2 and 3. Groups 2 and 3 demonstrated a significant reduction in wave I amplitude and increase in wave V/I ratio on ABRs compared with Group 1. No significant difference in latency in ABRs. No significant difference in DEOAEs among groups.
Suresh and Krishnan (2021) [68]	DPOAEs and ABRs (2 experiments): (1) ABR measured in a quiet setting from 30 dB to 90 dB, with a 10 dB stepwise increase between measurements (2) ABRs recorded with a 70 dB click stimulus with simultaneous broadband noise masking at 50, 60, and 70 dB	56 total participants. There were 28 normal-hearing adults who participated in a marching band for 5 years and 28 adults as part of a low-risk control group with no history of occupational exposure.	Conventional and high-frequency audiometry	No significant difference between groups in DPOAEs or PTA. In Experiment 1, the high-risk group showed smaller wave I amplitudes at moderate and high sounds compared with the low-risk group. Wave III and V amplitudes were similar, though the wave V/I ratio was enhanced in the high-risk group. Experiment 2 also demonstrated a smaller wave I amplitude for the high-risk group, though the relationship was less pronounced than in Experiment 1. Wave V amplitude and latency was similar between groups.
Bhatt et al. (2022) [69]	MEMR, DPOAE, ABR	30 healthy adults aged 18–35; 15 with high NEB and 15 with low NEB.	Conventional and high-frequency audiometry, SiN.	The high NEB group showed significantly reduced DPOAEs and ABR wave I amplitude compared with the low NEB group. The high-risk group also performed significantly worse on the SiN assessment. MEMR was not significantly different between groups, though it was associated with NEB.
Bramhall et al. (2022) [70]	MEMR	184 total participants, containing 92 young veterans and 92 non-veterans.	None	A 25% reduction in MEMR magnitude was found in the veteran compared with the non-veteran population. The reduction in MEMR is consistent with the decrease in wave I amplitude and EFR magnitude in the veteran population.
Cildir et al. (2022) [71]	ABR	2 groups separated based on lifetime noise exposure. High-risk (*n* = 39) and low-risk (*n* = 30).	Dichotic digit test, matrix sentence test, amplitude modulation detection test, loudness adaptation test	The high-risk group had a significantly lowered wave I amplitude compared with the low-risk group with 70 and 80 db stimuli. Compared with the low-risk group, the high-risk group demonstrated a significantly more rapid adaptation to stimuli in the presence of background noise (120 s vs. 40 s). Amplitude modulation detection performance was higher in the high-risk group. There was a significant difference in matrix sentence test performance between groups only in the right ear, though the authors do not deem this result significant.
Lai and Bidelman (2022) [72]	EcochG: measuring summating potentials (SPs) with paired versus single clicks	18 young adult subjects, between 23 and 33 years. Low- (*n* = 9) and high-risk (*n* = 9) groups demonstrated normal and elevated high-frequency thresholds, respectively.	Conventional and high-frequency audiometry, SiN, and Listening Effort and Noise Sensitivity Questionaire (LENS-Q)	High-risk subjects demonstrated significant increases in SP amplitudes in response to paired versus single clicks compared with the low-risk subjects, which had more consistent SP amplitudes. This finding was particularly relevant for paired click stimuli with short inter-click intervals. Larger SP ratios were correlated with a worse SiN performance and subjective hearing acuity.
Pinsonnault-Skavernina et al. (2022) [73]	ABR	80 total subjects, including 40 young adults with occupational noise exposure and 40 non-exposed young adults.	SiN	No significant difference in ABR measures between the noise-exposed and control group, including wave 1 amplitude, wave I/V ratio, and wave V latency shift. However, the noise-exposed group performed significantly worse on the SiN assessment.
Pinsonnault-Skavernina et al. (2022) [74]	ABR, EcochG, equivalent rectangular bandwidth (ERB)	40 total subjects, including 27 military recruits exposed to firearm/artillery noise and 13 non-exposed controls.	SiN	Compared with the control group, military recruits performed worse on the SiN test, had a higher ERB at 4 kHz, reduced wave I amplitude at 75 dB, and delayed wave V latency. No significant difference in ABR wave I/V ratio or EcochG measures, including summating potentials (SP), action potentials (AP), or SP/AP ratio.
Suresh and Krishnan (2022) [75]	Frequency-following response (FFR) of envelope periodicity (FFR_ENV_) and temporal fine structure (FFR_TFS_). Responses were evaluated in quiet and with background speech.	48 young adults aged 18–30. The high-risk group (*n* = 24) participated in a marching band for at least 5 years, and the low-risk group (*n* = 24) had low-exposure noise history.	None	No difference In neural encoding between groups for FFR_ENV_ or the F1 formant of FFR_TFS_ in noise and quiet conditions. Paradoxically, the high-risk group demonstrated enhanced representation of F2 harmonics in FFR_TFS_, though the authors suspect this may be due to music experience-dependent plasticity.
Aedo-Sanchez et al. (2023) [76]	ABR	45 total subjects, divided into risk groups by age: the young group (*n* = 27) and the adult group (*n* = 18).	Conventional and high-frequency audiometry, SiN	The adult group recorded significantly lower wave I and V amplitudes than the young group at suprathreshold levels (80 dB). Adults also exhibited delayed wave V latencies at threshold and supra-threshold levels. In terms of behavioral measures, adults had significantly worse tonal thresholds at high frequencies and worse performance on the SiN assessment.
Sabzinasab et al. (2023) [77]	ABR wave V latency with masking	A total of 38 males, with *n* = 20 experiencing occupational noise exposure and *n* = 18 without.	None	There was no significant difference between groups for wave V latency with masking conditions. However, there were significant differences within groups.
Vasudevamurthy and Kumar (2023) [78]	MEMR threshold and strength	50 total subjects; *n* = 25 individuals who were exposed to occupational noise for >1 year and 25 controls with no exposure.	None	MEMR strength was reduced in the noise exposure group compared with the control group. The MEMR threshold remained similar in both groups.
Yuan et al. (2023) [79]	DPOAEs, EcochG, ABR	101 young adults divided into high risk (*n* = 51) and low risk (*n* = 50) based on noise exposure.	Conventional and high-frequency audiometry, SiN	There were several differences between the high-risk and low-risk group. The high-risk group recorded lower DPOAEs at 8 kHz and 10 kHz, lower amplitudes of SPs and APs in EcochG, and a higher wave III amplitude. Speech discrimination scores were also significantly worse in the high-risk group.
Jamos and Rickman (2024) [80]	MEMR, EcochG, DPOAEs	21 young adult participants. High-risk (*n* = 11) and low-risk (*n* = 10) groups assigned based on noise exposure history.	SiN	The high-risk group exhibited worse performance on the SiN test, as well as smaller AP amplitude and greater SP/AP amplitude ratio in EcochG. The high-risk group also exhibited a higher probability for an elevated/absent MEMR threshold. There were no significant differences in hearing thresholds or DPOAEs at any tested frequency between groups.
Yaşar et al. (2025) [81]	EcochG: SP/AP ratio	68 people aged between 18–65 years. A symptomatic group (*n* = 35) of patients with a 2-month history of difficulty understanding speech in noisy environments and a control group (*n* = 33) of healthy volunteers.	None	The symptomatic group registered a statistically higher SP/AP ratio compared with the healthy control group.

## 3. Results

### 3.1. Criteria for Risk of HHL

With a total of 21 studies, there were a variety of different methods by which subjects were stratified into high- or low-risk groups to assess synaptopathy. A total of 16 studies utilized noise exposure as a means of assessing relative risk for HHL. Seven of these studies assessed long-term/lifetime noise exposure for group assignment, and the remaining nine studies used occupational exposure as the independent variable. Three studies used age to assess HHL, one of which utilized a tri-group design [67] studying the effects of occupational noise exposure and age together. Given that aging and noise exposure are two of the most prominently identified risk factors for HHL, the use of these measures to identify at-risk patients was an essential component for measuring synaptopathy [14,21]. Additionally, one study determined risk via the presence of elevated auditory thresholds at high frequencies [72], and another used the symptomatic endorsement of difficulty understanding speech in noisy environments [81].

### 3.2. Participant Characteristics

All participants demonstrated an auditory threshold of ≤25 dB up to 8 kHz, the standard frequency range. Besides having normal audiograms, participants varied significantly in terms of age, gender, and demographic risk factors for HHL. Several studies focused on young populations (<30 years old) with varying levels of lifetime noise exposure, or occupational exposure such as involvement in a marching band [68,75] or veteran status [66,70]. Other studies like Carcagno and Plack [63] used a larger, more heterogenous population, with age as the suspected risk factor for HHL.

### 3.3. Electrophysiological Measures

A wide variety of electrophysiological measures were present among the 21 studies, with several utilizing a battery of tests to assess synaptopathy. The most common test was the auditory brainstem response (ABR) test, measured in 15 studies. Though ABR analysis was frequently performed, varied data measures were extracted from each study. For example, many studies measured wave I amplitude, which reflects the synchronous firing of the auditory nerve and is correlated to the number of synapses between inner hair cells and auditory nerve fibers [82]. The wave V amplitude and the wave I/V (I/V) ratio were also frequently assessed; wave V reflects activity from the inferior colliculus, an aspect of the central auditory pathway, and the I/V ratio quantifies the integrity of the peripheral and central pathways [83]. Of the 15 studies measuring ABR, 10 studies found significant differences in ABR findings between groups, the most common of which was a wave I amplitude reduction followed by a varied I/V ratio.

Six studies utilized electrocochleography (EcochG), which measures electric potentials generated by cochlear hair cells and the auditory nerve in response to a sound stimulus [84]. Generally, the EcochG measures summation potential (SP), known as the electrical signals from hair cells, and the action potential (AP), the firing from the auditory nerve [85]. Four of the six studies found significant EcochG findings, including a decrease in AP amplitudes [42,79], inconsistent SP amplitudes [72], and an increased SP/AP ratio [80,81] compared with control groups. One study found no significant differences in SP, AP, or SP/AP ratios between groups [74].

Four studies measured the middle ear muscle reflex (MEMR), the contraction of the stapedius muscle in response to loud sounds [86]. This reflex is a supra-threshold response that relies on competent afferent conduction from auditory nerve fibers, hence its potential value in assessing synaptopathy [86]. Three of the four studies found abnormalities in the MEMR between groups, either in threshold or strength, with the fourth study [69] finding an association between noise exposure and MEMR response.

Two studies measured the frequency-following response (FFR), a reflection of the phase-locked neural activity to acoustic stimuli [87]. Temporal fine structure (TFS) and envelope following response (EFR) are components of FFR that were assessed in both studies. The former represents the ability to encode fine, rapid variations in carrier frequency, while the latter details the ability to track the slow, amplitude-modulated changes to sound [87,88]. The Carcagno and Plack study [63] found that TFS and EFR decreased with age, while Suresh and Krishnan [75] found no differences in neural encoding for these measures between a group of musicians and healthy controls.

Distortion product otoacoustic emissions (DPOAEs) were also measured among five studies, though their findings were contradictory. DPOAEs are an assessment of outer hair cell function, rather than neural function, and thus are not generally considered a direct proxy for measuring synaptopathy [89]. However, two studies found significantly reduced DPOAEs among the high-risk groups, while the remaining three studies found no significant differences.

### 3.4. Behavioral Measures

There were 13 out of the 21 studies that assessed behavioral measures of HHL. Just as with the electrophysiological measures, behavioral measures varied in terms of their significance between groups for each study. Perhaps the most consistent finding across several studies was that the high-risk group performed comparatively worse on the SiN assessment than the low-risk group. High-frequency audiometry was also measured in a few studies. For example, Aedo-Sanchez et al. [76] found that the high-risk adult group had significantly worse tonal thresholds at high frequencies. Audiometric thresholds at standard frequencies were measured across all studies to confirm normal audiograms.

### 3.5. Study Characteristics and Findings

Table 2 below provides information for each of the selected studies in this review. Information regarding the diagnostic methods, subject population, behavioral correlates, and key findings of each study are noted.

## 4. Discussion

### 4.1. Auditory Brainstem Response

Based on the results of this systematic review, it appears that the auditory brainstem response is both the most common method of diagnosis, as well as a relatively accurate test for assessing synaptopathy. A reduction in wave I amplitude appears to the be one of the most sensitive measures of synaptopathy, with 8 of 12 studies demonstrating a significant reduction compared with the control group. Wave I directly measures the direct neural integrity of inner hair cells and their ability to convey information to the auditory nerve [66]. The wave I/V (or V/I) ratio may also be a valuable measure of synaptopathy, comparing the integrity of peripheral and central auditory pathways [83]. Suresh and Krishnan [68] cited an enhanced V/I ratio among marching band subjects compared with controls, despite both groups registering similar wave V amplitudes. Thus, several authors suggest that HHL patients demonstrate a relatively increased central gain, as measured by wave V, as a compensating response for a reduced peripheral auditory stimulus due to synaptic hair cell damage, as noted by the wave I response [67,82]. A mice study similarly demonstrated hyperactivity in the central auditory pathway in mice with noise-induced synaptic loss [90]. Three of the five studies included in this review reporting a wave I/V ratio indicated discrepancies between groups in this value. Wave V latency was also commonly assessed among ABR studies, a measure of the timing of neural conduction through the brainstem [91]. However, only one study from Pinsonnault-Skavernina et al. [74] found a significant difference in wave V latency shift. Three studies also assessed ABR with masking conditions, in which responses were measured in the presence of background noise [92]. Given that symptomatic patients with HHL often endorse difficulty hearing in noisy environments, this added condition sought to elicit stronger discrepancies in ABR responses. However, none of the studies found significant differences in masking conditions, with Carcagno and Plack [63] interestingly demonstrating that masking elicited similar responses between groups in comparison with the non-masking condition.

### 4.2. Electrocochleography

Similar to ABR, electrocochleography also appears to be a sensitive measure of synaptopathy, with four of six studies finding group differences in EcochG. For example, Yuan et al. [79] found decreased SP and AP amplitudes among the high-risk noise exposure group compared with controls, while studies from Jamos and Rickman [80] and Yaşar et al. [81] found increases in the SP/AP ratios. The authors of Yuan et al. [79] claim that the SP amplitudes are reduced due to a decrease in the proportion of inner hair cell transduction channels from noise or age-induced damage. Jamos and Rickman [80] further assert that the SP/AP ratio is elevated due to the significant reduction in the AP amplitude, neural response generated by auditory nerve fibers, and an equivalent to the wave I ABR amplitude. Lai and Bidelman [72] took a slightly different approach to measuring EcochG, citing an increase in SP amplitudes in response to single and paired-click acoustic stimuli. Interestingly, these authors argue that SP must be measured to assess synaptopathy, and not the AP, because the AP response is “all-or-nothing” in nature and exhibits a refractory period. In contrast, the SP is a receptor potential that is not constrained by refractoriness. Synaptopathic patients potentially exhibit reduced excitatory postsynaptic potentials (EPSP) due to synaptic damage, thus causing an unopposed increase in SP when stimulated by paired clicks with small inter-click intervals [72]. Though this novel method of measuring synaptopathy was not assessed in other studies, it holds significant potential as a diagnostic tool, assuming ample reproducibility of findings can be achieved.

### 4.3. Middle Ear Muscle Reflex

The MEMR also holds potential as a diagnostic indicator for synaptopathy, though only 4 of the 21 studies included MEMR as a proxy of synaptopathy. With three of these studies indicating abnormalities in MEMR in between-group analyses, as well as the minimal specialized equipment required for measurement, MEMR has realistic potential for clinical use in identifying HHL. Mechanistically, synaptopathy has been theorized to affect the low-spontaneous rate (SR) fibers of spiral ganglion nerves, which are crucial for encoding sounds in loud environments [93]. These same SR fibers are important drivers for inducing the MEMR, thus serving as a potential indicator of synaptopathy [86]. In this review, however, there are a few discrepancies between studies in terms of MEMR outcomes among studies with significant outcomes. For example, Vasudevamurthy and Kumar [78] indicated a reduction in MEMR strength but not threshold among at-risk subjects, whereas Jamos and Rickman [80] found the high-risk group to have a higher probability of an elevated threshold or absent reflex. Thus, further studies into the intricate relationships between MEMR with respect to strength and threshold changes must occur before a true diagnostic value can be assessed.

### 4.4. Frequency-Following Response

Two total studies employed the FFR as a diagnostic method. Only one study, from Carcagno and Plack [63], identified significant findings related to FFR, demonstrating that TFS and EFR both decreased with age, thereby suggesting a decreased neural representation of sound in synaptopathic subjects. A recent study from Märcher-Rørsted et al. [94] confirms similar findings, claiming that age-related reductions in FFR amplitude are consistent with peripheral neural degeneration. In contrast, the results from Suresh and Krishnan [75] indicate no significant differences in TFS and ENV between groups. However, the authors note this may be confounded by the comparison of musicians and non-musicians as controls. With only two relevant studies to FFR in this review, more studies assessing synaptopathy are necessary to determine its efficacy as a diagnostic measure, though emerging experiments may be promising.

### 4.5. Other Synaptopathy Tests

Dewey et al. [64] was the only study in the current review to assess fMRI in relation to synaptopathy. The results from this study found higher levels of neural activity in central auditory pathways following exposure to broadband noise. The authors theorize that this may be due to hyperactivity of the central region as a compensatory mechanism for the synaptic loss of SR fibers in the peripheral pathway. Though this may be an effective means of identifying synaptopathy, it is likely not clinically practical to expect large volumes of patients to use an fMRI for diagnosis.

Though a few studies measured DPOAEs, they do not measure the integrity of synaptic connections and instead measure outer hair cell function [95]. Thus, DPOAEs should not generally be used to diagnose synaptopathy. Many studies also required normal DPOAEs in conjunction with normal audiograms for inclusion.

Several studies also employed behavioral measures of HHL, the most notable of which was the SiN assessment. SiN and other behavioral measures, such as the matrix test, were frequently found to be reduced in the high-risk groups compared with the low-risk groups. These measures are important in terms of determining symptomatic effects of HHL. However, because they are subjective and do not directly quantify synaptopathy, these findings alone do not provide proper evidence to diagnose HHL. A combination of electrophysiological and behavioral tests likely will improve diagnostic accuracy and provide a more comprehensive understanding of synaptopathy.

## 5. Conclusions

**Figure 2:**
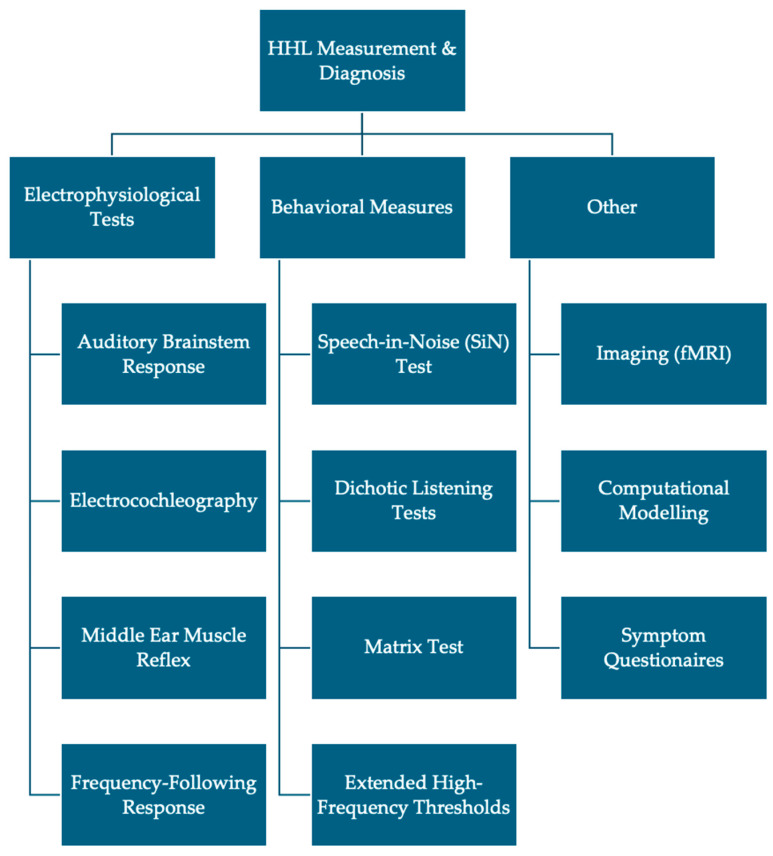
Common diagnostic techniques used to assess the presence of HHL/cochlear synaptopathy. Note: the figure does not include all diagnostic modalities.

This systematic review highlights the diverse and evolving landscape of diagnostic tools for hidden hearing loss (Figure 2). While significant progress has been made in recent years, the lack of standardization remains a key barrier to clinical implementation. Moving forward, a concerted effort to harmonize methodologies and incorporate multimodal diagnostic strategies will be essential for advancing our understanding and management of HHL. ABRs, in particular wave I amplitude and I/V ratios, were most commonly assessed in this review, with several studies identifying significant differences in these measures. Similarly, SP and AP amplitudes from EcochG appear to be sensitive to synaptopathy, though further studies comparing EcochG with ABR are necessary to determine which assessment is more sensitive. MEMR is a promising emerging method of diagnosis that requires comparatively simpler equipment than ABR and EcochG, providing a high potential for clinical utility and rapid screening for HHL. In contrast, studies assessing FFR may not be as reliable as the previously listed electrophysiological measures, though more literature surrounding FFR is necessary to determine its value.

A two-pronged approach combining electrophysiological and behavioral measures could prove useful in improving diagnostic confidence for HHL. Integration of these two measures can bridge the gap between neural dysfunction and perceptual abnormalities. Furthermore, this framework offers a gradient of impairment rather than a binary diagnosis, which may be useful in determining relative risk for more severe (audiometrically detectable) HL in patients already suffering from HHL. Generation of a standardized diagnostic methodology will allow for more consistency in measurements and enable earlier identification of subclinical auditory deficits.

## Data Availability

No new data were created.

## Supplementary Materials

The following supporting information can be downloaded at: https://www.mdpi.com/article/10.3390/diagnostics15060742/s1, The PRISMA Checklist.

## Abbreviations

The following abbreviations are used in this manuscript:

HL	Hearing loss
HHL	Hidden hearing loss
SGC	Spiral ganglion cell
ABR	Auditory brainstem response
PTA	Pure-tone audiometry
SNHL	Sensorineural hearing loss
SiN	Speech-in-noise
EcochG	Electrocochleography
SP	Summating potential
AP	Action potential
MEMR	Middle ear muscle reflex
FFR	Frequency-following response
TFS	Temporal fine structure
EFR	Envelope following response
DPOAEs	Distortion product otoacoustic emissions
NEB	Noise exposure background
SR	Spontaneous rate
EPSPs	Excitatory postsynaptic potentials
